# The Development of Tungsten Biochemistry—A Personal Recollection

**DOI:** 10.3390/molecules28104017

**Published:** 2023-05-11

**Authors:** Wilfred R. Hagen

**Affiliations:** Department of Biotechnology, Delft University of Technology, Building 58, Van der Maasweg 9, 2629 HZ Delft, The Netherlands; w.r.hagen@tudelft.nl

**Keywords:** tungsten, tungstoenzyme, EPR, tungstopterin, *Pyrococcus furiosus*, transport, metallomics, aldehyde oxidoreductase

## Abstract

The development of tungsten biochemistry is sketched from the viewpoint of personal participation. Following its identification as a bio-element, a catalogue of genes, enzymes, and reactions was built up. EPR spectroscopic monitoring of redox states was, and remains, a prominent tool in attempts to understand tungstopterin-based catalysis. A paucity of pre-steady-state data remains a hindrance to overcome to this day. Tungstate transport systems have been characterized and found to be very specific for W over Mo. Additional selectivity is presented by the biosynthetic machinery for tungstopterin enzymes. Metallomics analysis of hyperthermophilic archaeon *Pyrococcus furiosus* indicates a comprehensive inventory of tungsten proteins.

## 1. Introduction

In 1991, I was an Associate Professor of biochemistry at Wageningen University, NL, with my research focussing on the spectroscopy of metalloproteins. When my department head, Cees Veeger, asked me to setup an MSc course in the then very much emerging field of bioinorganic chemistry, I considered myself very lucky to have received a copy of the just published monograph “The Biological Chemistry of the Elements” by Fraústo da Silva and Williams [1], a book that abounded in concepts to transform the field from being descriptive into mechanistically interpretive. I did a simple conversion of these concepts from book pages into slides, which afforded a lecture series that was both coherent and popular with the students. In subsequent meetings over the years with Bob Williams at conferences and advanced courses, I had a chance to refine my understanding of those concepts, which now also form the basis of the present review.

**Figure 1:**
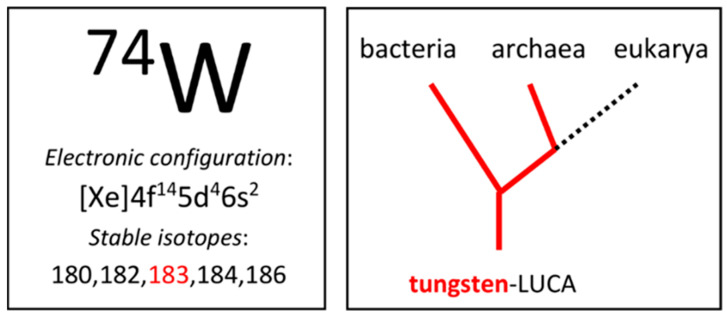
Tungsten in the periodic table and in the domains of life. One of the five stable isotopes has a finite nuclear spin, I = 1/2. Tungsten-LUCA is the last universal common ancestor with tungsten biochemistry.

A distinctly enjoyable and potentially informative aspect of present-day biochemistry, when seen as the result of cellular evolution, is the relevance of “the why question”, hence: why tungsten? In other words, given a certain environmental setting at a given period in time, what specific advantages can a cell, engaged in a survival-of-the-fittest struggle, expect to gain by opting to fish for, import, and metabolize a not very abundant third-row transition element and, in some cases, make its viability mandatorily dependent on the presence of that element? For the first step in this process, we can perhaps surmise that it is inherent to evolution that whenever an element is present, cells will inevitably try out its biochemical potential. For this action, general boundary conditions of abundance and availability have been formulated by Fraústo da Silva and Williams [1] in their 1991 monograph: (1) the element should have at least some occurrence in the earth crust, preferably in a not too irregular distribution; (2) the element should have a finite solubility in the solvent of life, that is, water. Element 74, tungsten (Figure 1), nicely fulfils these criteria; its occurrence is not rare and quite homogeneous, and its common speciation in water is the oxoanion, WO_4_^2–^, which is highly soluble. Remarkably, this obvious suitability was insufficient to convince Fraústo da Silva and Williams of the value of tungsten in modern-day biochemistry. Rather, they were of the opinion that evolution has decided that tungsten is on its way out of terrestrial life in favor of molybdenum: “tungsten is restricted to a few primitive or last remnants of primitive species and does not seem to be required by modern living organisms” (the quote is literally repeated in the 2001 second edition). To me, this seems to be a misconception of anthropocentric (or perhaps eukaryocentric) nature: to decide that contemporary species are primitive just because humans chose to call them archaea. Instead of making this choice, I think we should not deprive ourselves of the possibility to ask the question “why tungsten now”, which is particularly pertinent where closer congeners than W and Mo are hard to find in the transition-ions block. Our toolbox for this inspection contains discriminators (worked out in detail by Fraústo da Silva and Williams) like reduction potentials, Lewis acidity, ionic radii, and kinetics of ligand exchange, all in a biological setting.

## 2. EPR Is a Specific W-Monitor

Transition-ion-containing proteins are particularly suited for scrutiny by spectroscopy. An unwritten rule of thumb, let’s call it “the proper sequence rule of spectroscopies”, says that we first turn to UV-vis spectroscopy due to its relatively low cost, ease of operation, and ready availability, and subsequently try our luck with EPR spectroscopy, which is not so cheap, not so easy, and less readily available. Most other forms of spectroscopy typically enter consideration at a later, more advanced stage of our research [2,3]. UV-vis spectroscopy may suffer from low spectral resolution and, in the case of Mo- and W-pterin systems, from low intensity next to the absorption of iron-sulfur clusters, which are also contained in the vast majority of the enzymes in this class. So EPR jumps to place one, which makes it worthwhile to head-on consider the idiosyncrasies of tungsten EPR in some detail. In addition, I should not of course, deny that doing EPR has been one of my favorite pastimes ever since my days as an undergrad in the mid-seventies in the lab of Siem Albracht in Amsterdam.

Tungsten is W^6+^ in the environment of cells, and in their interior, reduction can take place to W^5+^ and W^4+^. The ions’ respective outer shell electron configurations are 5d^0^, 5d^1^, and 5d^2^. The closed-shell d^0^ system is diamagnetic and has no EPR. The d^2^ system can be S = 0 or S = 1, where the latter is less likely in the lower regions of the periodic table and also in the semi-flat ligand field provided by the two pterin ligands. EPR, or more generally, paramagnetism, has never been reported for W^4+^ in biological systems. Thus, we are left with the W^5+^, d^1^ system with S = 1/2.

**Figure 2:**
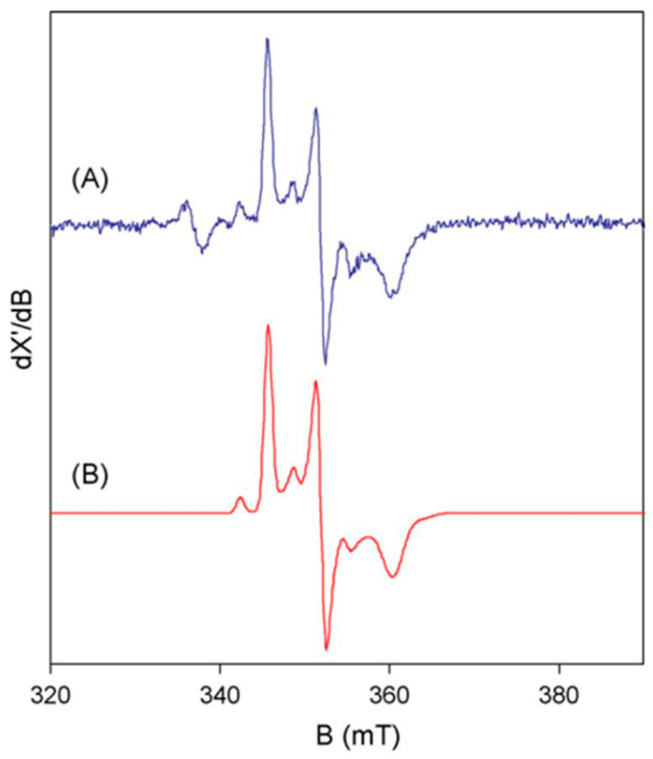
The basic features of tungsten(V) EPR from tungstoenzymes. The spectrum (**A**) is from *Pyrobaculum aerophilum* aldehyde oxidoreductase, AOR. The simulation (**B**) is based on S = 1/2 with three g values all <2 and three hyperfine values for 14% ^183^W with I = 1/2 [4].

Basic EPR theory dictates that, for d^1^ systems (e.g., Ti^3+^, V^4+^, Mo^5+^, W^5+^) of axial or lower symmetry, spin-orbit coupling within the ground d-manifold of states results in all g values ≤ 2. For the low symmetry W^5+^-pterin site in proteins, we expect g_z_ ≠ g_y_ ≠ g_x_, that is, three distinct features in the EPR first-derivative spectrum each at g < 2. Furthermore, W comes with five stable isotopes, each with a nuclear spin of I = 0, except for ^183^W, which has I = 1/2. The latter will split each of the three g features in two, with splittings A_z_ ≠ A_y_ ≠ A_x_, and since the natural abundance of ^183^W is circa 14%, the relative intensity of the split spectrum with respect to the un-split one from the other isotopes will be 100 × [(14/2)/86] ≈ 8%. The resulting pattern (see the example in Figure 2 [4]) is unique and should be a straightforward identifier of W^5+^ in complex EPR spectra. An additional identifying criterion comes from the relatively slow spin-lattice relaxation rate of d^1^ systems, which makes it possible to detect W(V) signals without much broadening up to ambient temperature, providing a clear discriminator against the EPR of iron-sulfur clusters.

## 3. W-Enzymes on the Horizon

As an orientational aide, I summarize classes of W-enzymes and their abbreviations in Table 1, which is a modification of Scheme 10.1 in [5]. In the early seventies, long before I even became aware of the biological relevance of tungsten, microbiologist Jan Andreesen, following the completion of his PhD in Göttingen, moved to the University of Georgia in Athens to do two years post doc with Lars Ljungdahl. They studied sugar fermentation in the strict anaerobe *Clostridium thermoaceticum* (in 1994 renamed as *Moorella thermoacetica*), and they focussed on the enigmatic NADP^+^-dependent enzyme formate dehydrogenase (FDH) catalyzing the reaction CO_2_ + NADPH ⇆ HCOO^−^ + NADP^+^. It was found that FDH activity increased, especially with co-administration of selenite, both by addition to the growth medium of molybdate and of tungstate, and moreover that the stimulation was additive, be it that the contribution of W was clearly superior over that of Mo [6]. In my view, tungsten bio(inorganic) chemistry was born then, although the authors were careful to note that they did not yet know whether W was in the active site or only stabilizing the enzyme. Later, the matter was settled when W was established to be an intrinsic part of active enzyme [7,8] but, remarkably, discussion of the additive effect of Mo and W on activity appears to have since fallen by the wayside.

**Figure 3:**
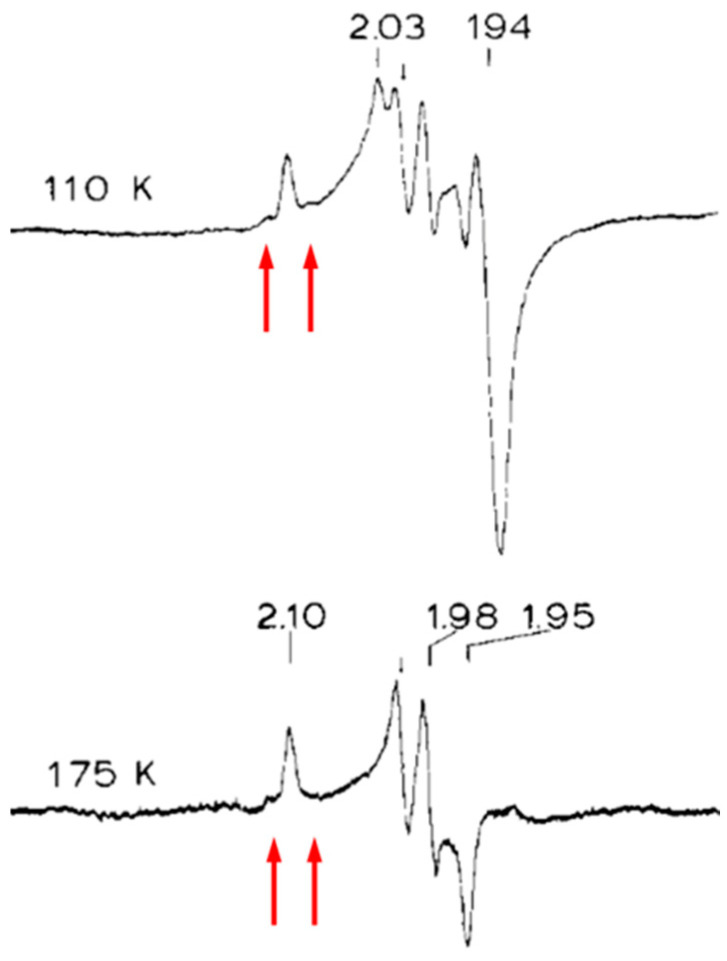
Early EPR study of a tungstoenzyme. Formate dehydrogenase, FDH, from *Moorella thermoacetica* was poised at −450 mV. The high-temperature signal was ascribed to W^5+^, however, one of the g values is >2 in contradiction to basic EPR theory. Arrows indicate where I believe I observed ^183^W hyperfine lines. Reproduced from [10] with permission from Elsevier.

In a perhaps somewhat overconfident attempt to jump “the proper sequence rule of spectroscopies” Stephen Cramer opened spectroscopic studies on *M. thermoacetica* W-FDH with L_III_-EXAFS, and his conclusion was, not unexpectedly, that the details of the W coordination remained uncertain [9]. Then Ed Solomon and colleagues stepped back to follow the above-cited rule of thumb and applied EPR to *M. thermoacetica* W-FDH poised at low redox potential of −450 mV to stabilize W^5+^ [10]. Surprisingly, in their interpretation, the resulting spectrum was not unequivocally ascribable to tungsten. It exhibited three sharp g-value features alright, and it was readily observable up to 175 K (in contrast to the also present Fe/S cluster signals), but of the g-values, 2.10, 1.98, and 1.95, one was well above 2, in defiance of the basic g ≤ 2 rule for d^1^ systems. The authors did not consider ^183^W hyperfine splitting, although I am pretty confident that I can spot some of the satellite lines in their spectra (Figure 3).

Instead, they concluded that isotopic substitution studies were required, which unfortunately never materialized for this enzyme.

All in all, we have started the history of EPR W-monitoring in tungsten enzymology not with a solution but with a problem: there is a W-like signal, but one of its g values defies simple EPR theory. Note that several researchers have found ways to substitute W for Mo in certain molybdenum enzymes, resulting in a total loss of activity. In those cases in which EPR was detected from a tungsten signal, it exhibited normal spectroscopic properties in all respects, in particular g ≤ 2 [11,12]. Interpretation of unusual spectroscopic features is preferably done in the framework of a 3D molecular structure. Unfortunately, many more metalloproteins have been scrutinized spectroscopically than crystallographically, and *M. thermoacetica* W-FDH is a case in point. Until this day, the enzyme has never been crystallized, and its unusual EPR signal still hangs in the air as a spectroscopic orphan after 36 years.

Following up on the *M. thermoacetica* W-FDH in the first half of the 90s, several W-enzymes were isolated and purified to homogeneity from Clostridia in the lab of Helmut Simon in München [13,14], from methanogenic archaea in the lab of Rudolf Thauer in Marburg [15,16], and from hyperthermophilic archaea in the lab of Michael Adams in Athens, GA [17,18,19,20,21]. All enzymes exhibited some form of aldehyde oxidoreductase activity. Notably, seven out of these eight enzymes were from (hyper)thermophilic species. In addition, two out of eight have subsequently been crystallized, which leaves six of them as spectroscopic orphans.

## 4. The First X-ray Structure

**Figure 4:**
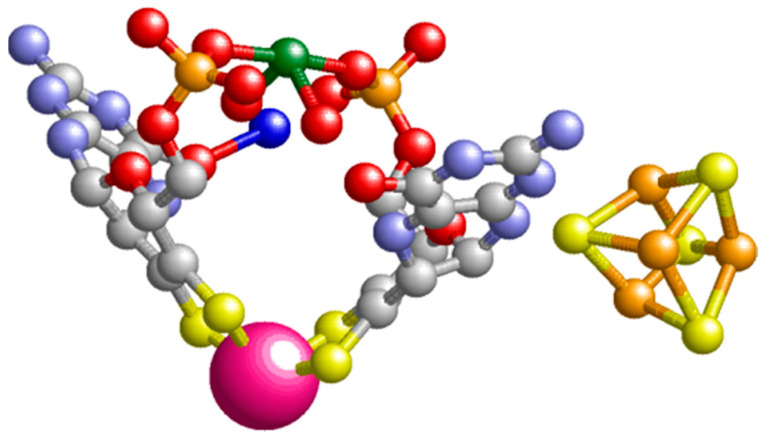
The prosthetic groups of *Pyrococcus furiosus* aldehyde oxidoreductase, AOR. The tungstopterin and the [4Fe-4S] cofactor are rendered in ball-and-stick display except for the tungsten ion (deep pink), which is in spacefill display. The green ion is magnesium, which couples the two pterin ligands via their phosphate side groups. The figure is based on pdb file 1AMF [23].

In the summer of 1994, I attended a Gordon Research Conference on Nitrogen Fixation in New London, NH, where I presented a talk with the hindsightedly somewhat ominous title “P-cluster EPR: Dissecting a Can of Worms”, on the multiple redox transitions in the 8Fe P-cluster of nitrogenase observed by my graduate student Antonio Pierik [22]. Douglas Rees from CalTech, Passadena, CA, USA, was also participating, and was scheduled to lecture on “Structures of Nitrogenase Proteins”. Uncharacteristically, Doug made a somewhat distracted impression on me, but matters quickly resolved when, near the end of his talk, he revealed his eagerness to give us an encore on a completely unrelated subject. A preliminary picture of the active-site structure of *P. furiosus* W-AOR (aldehyde oxidoreductase) appeared on the screen, thus attesting to the fact that a first example from the tungsten-molybdenum enzyme family was under crystallographic scrutiny. Three observations were particularly noteworthy: (1) the pterin ligand to W was modified by an intramolecular cyclization into a three-ringed structure: a pyranopterin; (2) two of these pyranopterins coordinated W by a total of four sulfur ligands (Figure 4); and (3) the enzyme contains a single [4Fe-4S] cluster. Two months later, the joint groups of Doug Rees and Mike Adams submitted a paper to Science on the complete 2.3 Å structure of the protein. Its appearance in the press half a year later [23] had the effect of a fog clearing, as now both the fields of Mo-enzymes and W-enzymes were provided with an initial framework for the interpretation of functional and spectroscopic observations, although consistency of this interpretation is not necessarily guaranteed by the availability of a structure, as later transpired for the *P. fusiosus* AOR and for other W-enzymes (see below).

## 5. My Chance Entrance into W Biochemistry

In 1984, after a two-year postdoc on bio-spectroscopies with Dick Dunham and Dick Sands at the University of Michigan in Ann Arbor, I arrived at Wageningen University and subsequently went through the usual academic ranks while I tried to build up a research group focussing on biochemistry and spectroscopy of metalloproteins. Late in 1994, microbiologist Theo Hansen from Groningen University contacted me with a request to scrutinize an enzyme with EPR. His graduate student Charles Hensgens had isolated the protein from the sulfate reducing model organism *Desulfovibrio gigas* (also *Megalodesulfovibrio gigas*), grown on a tungsten-containing medium, as an aldehyde oxidoreductase [24]. Gram-negative bacteria were known to be able to metabolize molybdenum but not tungsten; this enzyme contained circa one W in addition to four Fe/S per subunit of a homodimer [25]. This was only the second bona fide, that is, active tungsten enzyme (not a molybdenum enzyme in which Mo was substituted with W, resulting in inactivity) looked at in EPR, and, remarkably, the unusual g > 2 value previously observed in *M. thermoacetica* W-FOR was absent: all g values were <2 as they were expected to be for d^1^ systems. Another noteworthy observation was that the EPR spectrum consisted of multiple W^5+^ signals, a phenomenon that was to be observed repeatedly in later studies on W-proteins. Yet another significant finding was that reduction of the enzyme with 1 mM dithionite at pH 7.5 led to the complete disappearance of the W^5+^ signals, indicating the ready attainability of the W^4+^ oxidation state. Unfortunately, no follow-up studies ensued, and the protein has never been crystallized.

## 6. A Decade of Studies on W-Enzymes from *P. furiosus* and Other Species

Around the same time, in the mid-90s, I decided to make hyperthermophilic biochemistry a new line of our group’s research. Having previously spent many hours as a young researcher in cold rooms to prevent enzymes from denaturating during purification, I was fascinated by the idea that there are also enzymes out there that appear to remain perfectly active at 100 °C. We decided to go for the extremophile *P. furiosus*, which at 90–95 °C had a welcome “*E. coli*-like” doubling time of 20 min and which had become a de facto biochemical model system for hyperthermophilicity, in large part due to the studies carried out in the early 90s in the Athens group of Mike Adams. Fortunately, Adams’ group had left more than enough loose ends for us to catch up and develop into friendly competitors. Tungsten enzymes in *P. furiosus* became one of the main sub-directions, and we started with the W-AOR, whose structure had recently been described [23]. We tried to solve a puzzle that emerged from studies by the Adams group: they initially purified *P. furiosus* AOR in an inactive form called the red tungsten protein (RTP), whose quite complex EPR was interpreted as stemming from an S = 3/2 system spin-coupled to another center of undetermined spectral parameters, while no putative tungsten signals were found [17]. A year later, they published a strict anaerobic isolation procedure resulting in an active enzyme, but they claimed that the EPR was identical to that of the inactive RTP [18]. Contrarily, graduate student Alexander Arendsen and I found that the EPR of active AOR was simpler than that of RTP: a [4Fe-4S]^1+^ gave rise to a not uncommon mixture of an S = 3/2 and an S = 1/2 signal, with no sign of spin coupling. Additionally, oxidation at a relatively high potential of +180 mV led to a W^5+^ signal with all g < 2, with characteristic ^183^W hyperfine splitting, and with a maximum intensity of 0.2 spins per monomer. Moreover, a much weaker signal with reduced g anisotropy appeared at intermediate potential [26]. Following a nomenclature later introduced by Mike Johnson and collaborators [27], we can conveniently name these spectral components the ‘high-potential species’ and the ‘mid-potential species’. The substrate crotonaldehyde caused the disappearance of the tungsten signals, consistent with reduction to W^4+^ [26].

Shortly after the appearance of our paper, the groups of Mike Johnson and Mike Adams reported the detection of another tungsten species in *P. furiosus* AOR, which titrated from W^6+^ to W^5+^ and to W^4+^ at subsequent potentials −365 and −436 mV, and which they therefore named the “low-potential species”. Its maximal spectral intensity corresponded to circa 0.23 spins per chemically determined tungsten [27]. Their conclusion that the low-potential species is associated with activity and the other two signals are from inactivated enzymes will be evaluated in Section 7.

We also analysed the EPR of two other *P. furiosus* W-enzymes, glyceraldehyde-3-phosphate oxidoreductase (GAPOR) and formaldehyde oxidoreductase (FOR), whose purification was previously reported by the Adams group [21,28]. In view of the sequence homology with AOR, it was perhaps no surprise to find multiple W^5+^ signals with ^183^W hyperfine splitting and S = 3/2 plus S = 1/2 mixture signals from a single [4Fe-4S]^1+^ cluster [29,30,31]. A fourth enzyme, called W oxidoreductase number 4 (WOR4), was reported by Adams’ group, but no W^5+^ or reduced [4Fe-4S] signals were found. Instead, a signal reminiscent of [4Fe-4S]^3+^ containing high-potential iron proteins, HiPIPs, which occur in photosynthetic microorganisms, appeared upon incubation with the strong oxidant hexachloroiridate [32]. The observation was, and still is, not understood. We have never succeeded in purifying WOR4. We did purify yet another W-enzyme, whose structural gene was already named *Wor5*, that is, encoding a fifth W oxidoreductase. We found WOR5 to be a wide-spectrum aldehyde oxidoreductase with high affinity for several substituted and non-substituted aliphatic and aromatic aldehydes with various chain lengths. The EPR was rather simpler than that of the previous *P. furiosus* W-enzymes: only one W^5+^ signal and only one S = 1/2 [4Fe-4S]^1+^ signal. The translated protein sequence suggested the cluster to be coordinated by three Cys and one Asp [33], which was recently confirmed in crystallography [34].

As a research sideline, we also looked at W-enzymes from other species, in collaboration with other groups: two formate dehydrogenases from *Syntrophobacter fumaroxidans* with the group of microbiologist Fons Stams in the next building in Wageningen [35]; DMSO reductase from *Rhodobacter capsulatus* with the groups of Susan Bailey in Daresbury and of David Garner in Nottingham [36]; and AOR from *Pyrobaculum aerophilum* (Figure 2) with the groups of Imke Schröder at UCLA and of Simon de Vries, next door in Delft [4]. The latter paper has added spiritual value: although Simon and I go back six decades from wild high school years, via chemistry classrooms, to parallel PhD projects at the University of Amsterdam, and years later became next-door colleagues once more when I gave up my double professorship at Wageningen and Nijmegen University and moved to Delft in 2000, and although we were long-term daily sparring partners on all aspects of science and the meaning of life, we never had any joint publication, except for this detour W-enzyme paper. After Simon unexpectedly passed away in 2015, I spend well over a year of full time research effort, to reconstruct what he had been doing in terms of building state-of-the-art equipment for ultra-fast enzyme kinetic studies, so that eventually his creations, consisting of a continuous-flow optical spectrometer with a dead time of 4 microseconds, and a rapid-freeze setup with a dead time of 80 microseconds [37,38,39], were ready to be taken over by my scientific successor Peter-Leon Hagedoorn and his dedicated technician Marc Strampraad, who in this very time span try their luck on pre-steady-state kinetics of W-enzymes, where conventional kinetic equipment proved to be too slow for turnover resolution.

## 7. Redox Thermodynamics of Tungstopterin Cofactors

As tungsten enzymes catalyse electron-pair redox conversion of organic substates—with the exception of acetylene hydratase—it would appear obvious to relate this activity to the redox steps associated with W^6+^, W^5+^, and W^4+^ in the active sites. Reductive charging of these sites can be affected by two subsequent e-loadings via single-electron transferring iron-sulfur clusters, consistent with the finding that several W-enzymes have only a single cluster per tungstopterin. Additionally, the relatively low reduction potentials of the W transitions appear to nicely align with the low E_m_^0′^s of the substrate/product couples. Thus, to complete the thermodynamic part of the enzymology, only some details remain to be filled in, regarding association-dissociation of substrates and products, pH dependence of E_m_^0′^s, and their associated coupled proton-electron transfer, and temperature dependence, particularly for (hyper)thermophilic enzymes. Experimental confirmation of this straightforward model, however, is not convincingly obtained on the basis of the available data.

As an initial vantage point, it is perhaps useful to realize that the data base is of limited size: a total of only six papers report on the reduction potentials of W in W-enzymes, two of which are from the Adams/Johnson groups [27,40], and the other four are from my group [26,30,31,36]. Additionally, the subject has not really raised general interest: our last report is from 2006 and no new data have been presented over the last 17 years. Furthermore, the data set appears to contain several internal inconsistencies. Bell-shaped EPR intensity versus potential curves for W^5+^ in the sequence W^6+^ → W^5+^ → W^4+^ were found for *P. furiosus* AOR [27], *P. furiosus* GAPOR [30], and *T. litoralis* FOR [40]. However, in all cases, there were also additional W^5+^ signals whose redox sequence was limited to W^5+^→ W^4+^. We initially reported the EPR from these mid-potential and high-potential species in *P. furiosus* AOR to be reactive towards the substrate crotonaldehyde [26], suggesting possible catalytic relevance or at least excluding their association with irreversible inactivation. Contrarily, Johnson et al. subsequently found the signals to appear in the spectrum of an O_2_-inactivated enzyme, and they also found anaerobic oxidation to +300 mV to result in loss of activity, whence their suggestion that at least the high-potential species must be associated with irreversible enzyme inactivation [27]. Later, they found a similar set of signals in *T. litoralis* FOR, but now all signals reacted reversibly in redox titrations [40]. In *P. furiosus* FOR, we found a mid-potential species in EPR-monitored redox titration, but a W^5+^ signal, identified as stemming from a low-potential species because it appeared upon enzyme incubation with either formaldehyde or dithionite, never showed up in the dye-mediated redox titration [31]. In summary, the putative significance of all these signals/species was not unequivocally established by 2006, and this continues to be the case up to this time.

Doug Rees’s 1995 paper on the structure of *P. furiosus* AOR carries the somewhat cryptic remark that “the arrangement (namely, one Cys ligand of the 4Fe/S cluster being in H-bond forming distance from the pterin N-8) suggests that the pterin ligand does not merely play a passive structural role but may be an active participant in the redox chemistry of AOR” [23]. One can read this as: “the pterin can act as a medium for facile e-transfer from the cluster to the tungsten”, but it can also be read as: “the pterin (after all, a quinone derivative) may act as an intermediary redox group by itself”. This latter possibility can be visualized in more than one way. An extreme view (model-3 in [2]) would be to assume that all redox chemistry is localized on the pterin and that the W never changes redox state. Since the electrons enter the enzyme via a single e-donating Fe/S cluster, this would imply that the pterin behaves equivalently to flavin, that is, to be protein-stabilizable as a radical with a single unpaired electron. However, observation of a radical EPR signal of significant intensity has never been reported in the W-enzyme literature. Another possibility (model-4 in [2]) would be that the pterin takes up two electrons (nearly) concomitantly, one from the Fe/S cluster and one from W^4+^, whereupon the latter turns into W^5+^, which has to be re-reduced by an electron from the re-reduced Fe/S. Yet another, very different possibility has been proposed for BCR (AOR family member) based on QM/MM explorations: a W^4+^ to W^5+^ oxidation creates a radical on the substrate, which is subsequently fully reduced by an electron from the pterin, whereupon the latter forms another radical [41,42]. But then again, radicals remain to be found in turning over W-enzymes. In summary, paper, or its electronic equivalent, is patient: many models can be jotted down, but their serious testing is still beyond our present horizon due to a paucity of relevant experimental data. Taking together all of the above, there is no escaping the conclusion that our understanding of the tungsten redox chemistry of the AOR family of W enzymes still leaves much to be desired.

**Figure 5:**
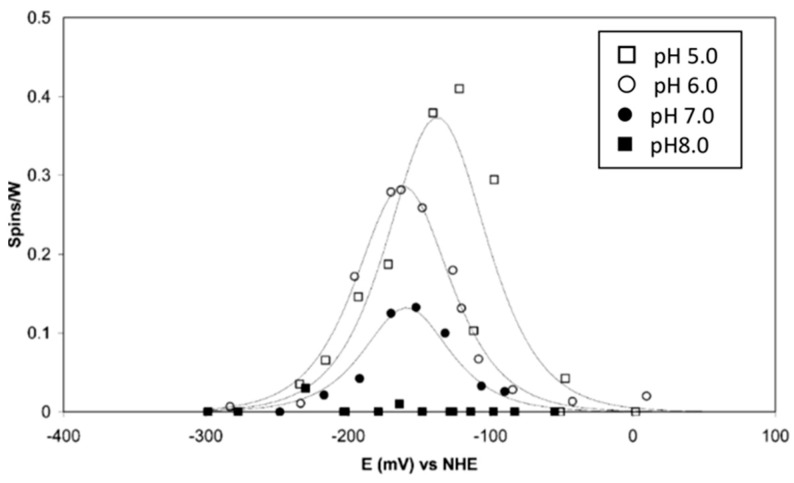
EPR monitored redox titrations of tungsten(V) in *Rhodobacter capsulatus* DMSO reductase. The titrations were done at the four indicated pH values. The reduction potentials, determined from fits to the Nernst equation, reveal an increasing cross over of E^0′^(W^6+^/W^5+^) and E^0′^(W^5+^/W^4+^) with increasing pH, resulting in a drop in maximum EPR intensity [36].

Perhaps the least controversial report on W-redox in W-enzymes is the joint publication of our group with those of Sue Bailey and Dave Garner on *R. capsulatus* DMSOR, which is a member of the DMSOR family of Mo/W enzymes, that is, not of the AOR family of exclusively W enzymes [36]. Peter-Leon Hagedoorn found a W^5+^ signal with splitting from a single proton and an equivalent signal without proton splitting, with their intensity ratio being a function of pH. The signals follow a bell-shaped titration curve for W^6+^ → W^5+^ → W^4+^, which is also pH-dependent (Figure 5).

Fitting of the titration curves to the Nernst equation reveals that the E_m_^0′^s of the two transitions are approximately equal at a pH of 5, but they increasingly “cross over”, i.e., E(W^6+^/W^5+^) < E(W^5+^/W^4+^) with increasing pH, which means that the maximum intensity of the W^5+^ signal reduces with increasing pH. At a pH of 8, the signal intensity has dropped to a level at which titration can no longer be monitored, as the extrapolated potential cross over difference is of the order of 100 mV. A generalized implication of this observation is that W^5+^ EPR signals from W- enzymes may be of impractically low intensity due to potential cross over. The prosthetic group begins to behave as a true electron-pair-accepting redox group, which we may conjecture to be advantageous from an enzymological point of view.

The E(W^6+^/W^5+^) has a slope of circa 30 mV per pH unit, while the E(W^5+^/W^4+^) is independent of pH. The mechanistic implication is that the uptake of the first electron is an example of a coupled electron-proton transfer (CEPT) reaction. Another mechanistic implication follows from the observation that the E-values at pH 7 are some 335 mV lower than the equivalent values for the Mo-DMSOR from the same species: the W-DMSOR is a much (17×) better DMSO reductase than the Mo-DMSOR, but, in contrast to the Mo enzyme, its specific activity for DMS oxidation is extremely poor [36].

## 8. Pre-Steady-State Kinetics

The holy grail of enzymology is a mechanistic understanding of an enzyme’s action under given environmental boundary conditions (temperature, pH, etc.). Ideally, this understanding encompasses a complete description, at the molecular level, of all enzyme-substrate intermediates, and their transformation rates. Their experimental identification requires resolution of a single turnover, in which all enzyme molecules act coherently in time. Specifically, apparatus is required that mixes (say, enzyme and substrate) and measures with a time resolution much superior to a single turnover time. In practice, approach to descriptional completeness may require many years of study by multiple groups, using a plethora of techniques. Such an extended activity may result in what we consider to be “a good understanding” of an enzyme. As an example, we say that we understand the enzyme cytochrome P450 “well”, because, over time, we think that we have identified its rate-limiting step(s) as well as its main intermediates, so that we can give a detailed description of its catalysis, perhaps comparable in quality to the description of reaction mechanisms in organic chemistry (e.g., [43,44,45]). Tungsto-enzymology is eons away from such a “well” understanding.

**Figure 6:**
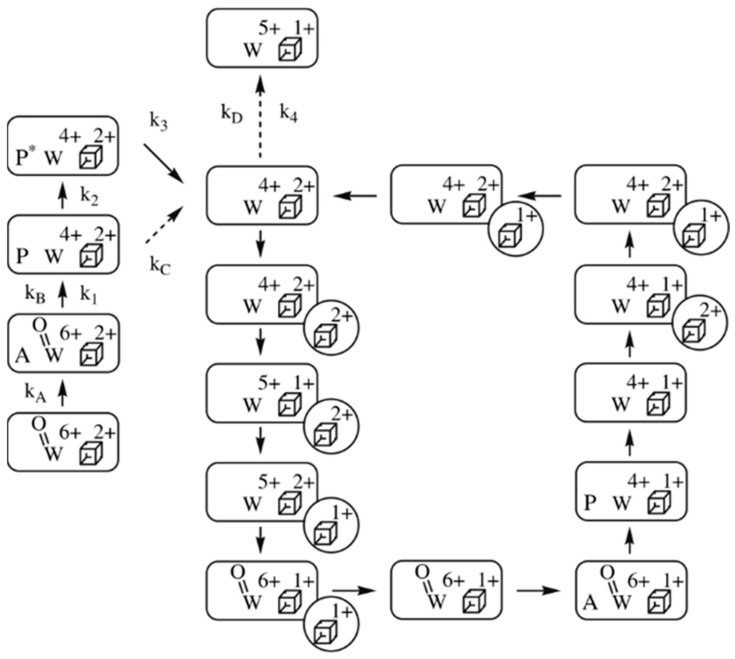
Reaction scheme of *Pyrococcus furiosus* formaldehyde dehydrogenase, FDH. The reaction is with substrate formaldehyde (symbol A) and electron donor ferredoxin (circle with a Fe/S cluster). P stands for product, and P* is ready-to-leave, deactivate product. The scheme shows a circular path for catalysis and linear side branches for activation/deactivation [46].

The specific activity of *P. furiosus* FOR with substrate formaldehyde at 50 °C and pH 8.4 is circa 20 s^−1^ [46]. Under these conditions we would thus have maximally 20^−1^ = 0.05 s to identify at least the rate-limiting step in catalysis. The homotetrameric FOR contains a single [4Fe-4S] cluster, in addition to the tungstopterin cofactor, per subunit, and the UV-vis spectrum is dominated by the absorption of the cluster. However, from studies of the DMSOR, which only contains the tungstopterin cofoactor, we know what its relatively weak (ε_max_ ≈ 1–2 mM^−1^cm^−1^) absorption spectrum looks like. My graduate student Emile Bol was indeed able to single out tungstopterin absorption in FOR from the development of optical spectra in time, in a stopped-flow spectrometer, following incubation of the enzyme with formaldehyde [46]. This then allowed for pre-steady-state kinetic analysis based on monitoring at selected wavelengths. In combination with data from steady-state kinetics with formaldehyde and with electron donor ferredoxin, analysis culminated in the at first sight rather involved scheme reproduced in Figure 6.

The first thing to notice is that the scheme is not a pure cycle, but a combination of a catalytic cycle and linear branches, where the latter represent activation/inactivation processes. A second remarkable fact is that individual reaction constants are only reported for the linear branches and, moreover, that the values for these constants are all less than the turnover rate. In other words, what apparently has been measured in pre-steady-state kinetics is not catalysis, but relatively slow activation of the fully oxidized enzyme through binding and oxidation of substrate. The catalytic cycle itself is only constructed on the basis of logically combining individual electron-transfer reactions. It is, furthermore, a rather primitive scheme, as it does not use any structural details of proteins and substrates, or any thermodynamic details such as (de)protonations. When one, on top of all this, realizes that this 2008 paper is until now the only pre-steady-state study of any tungstoenzyme, then the disconcerting conclusion becomes unavoidable that our enzymological knowledge of tungsten-based catalysis is virtually non-existent.

In the meantime, theoretical chemists have proposed different reaction mechanisms for tungstoenzymes, typically based in large part on static crystallographic data [41,42,47,48]. As an enzymologist, my advice would be: do not walk too fast; in the absence of proper experimental kinetic data, reaction-mechanism proposals have low testing potential and, therefore, limited relevance. In a positive vein, though, one can hope that deepening our knowledge of activation and deactivation processes may eventually be related to the multitude of observed W^5+^ EPR signals and their multiple redox responses. Clearly, much still remains to be discovered (and interpreted).

## 9. Getting W into the Cell

Is there anything special (selective) about tungstate import into cells? Oxoanions are internalized by means of ABC transporters, which typically consist of a soluble periplasmic binding protein-A, a dimeric transmembrane protein-B, and two membrane associated cytoplasmic ATPase proteins-C, to provide energy for active transport. In *Escherichia coli*, ModABC takes care of the import of molybdate, but it has equal affinity for tungstate (not used by *E. coli*) [49], which is perhaps not surprising since the two oxoanions have identical radii. *Eubacterium acidaminophilum* (now *Peptoclostridium acidaminophilum*) also has a molybdate-binding protein that cannot discriminate between molybdate and tungstate [50]. However, it also makes a protein called TupA (tungsten uptake protein-A) from a *tupABC* operon that has at least a 1000-fold higher affinity for tungstate than for molybdate [51]. The absolute affinity for tungstate, though, is rather low, with K_D_ = 500 nM. So, what about the archaea that do not use molybdenum but are mandatorily dependent on tungsten? Initially, it was suggested that their environment was simply high in tungsten [52], which would suggest that transport specificity would not be required. However, reported concentration numbers for Mo and W were mutually contradictory, partially unverified, and, moreover, did not apply to the shallow submarine thermal spring habitats of the model organisms *P. furisosus* [53] and *T. litoralis* [54,55], for which no environmental data were available.

So, it was only natural for my graduate student Loes Bevers to ask, well, so what about W uptake in *P. furiosus*? In its genome, she found an ABC operon with limited sequence homology to *E. coli*’s *modABC*. She named the system *wtp* (tungsten transport protein), and she cloned and expressed *P. furiosus wtpA* in *E. coli* and purified the protein WtpA to homogeneity [56]. In isothermal titration experiments, its affinity for tungstate was found to be some 30,000× higher than that of *E. acidaminophilum* TupA. The ratio of tungstate over molybdate affinities for WtpA was circa 650. How are we to know the significance of these numbers when the microbiologists, who isolate species like *P. furiosus* and *T. litoralis*, have never made a habit of also carrying out an elemental analysis of their water samples [53,54,55,57,58]?

**Figure 7:**
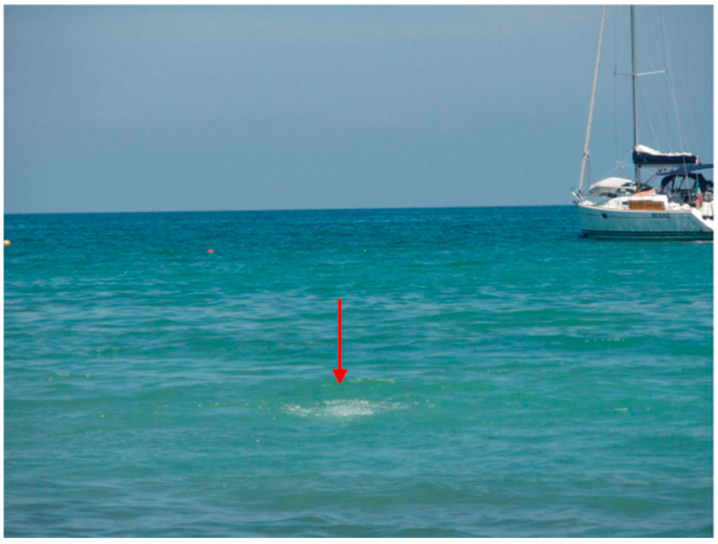
Outflow of hot gas bubbles from a marine hydrothermal vent. The vent is in shallow (circa 1.4 m) water in the Tyrrhenian Sea off the coast of the island of Vulcano, Italy.

In June 2009, I spent a holiday with my wife and two friends in Sicily. I convinced them that we really should take the ferry and spend a day on the island of Vulcano. Once arrived, the other three enjoyed the beach life, while I was trying to make up my mind where approximately Fiala and Stetter in 1986 had isolated *P. furiosus*, whose location they simply described as “marine sediments at the beach of Porto di Levante” [53]. I settled for a spot (Figure 7).

(38°25′01″ N 14°57′39″ E) on the sea floor at circa 1.4 m depth, which turned out to be only 1.5″ to the north of a position that was later, in 2017, reported by Stetter and many colleagues as the sampling place for their study on the diversity of bacteria and archaea from hydrothermal vents of Vulcano [58]. Armed with an empty plastic half liter bottle from a well-known dilute phosphoric acid containing soft drink brand, I managed, after some diving trials, to fill the bottle with water directly emerging from the local vent. Later, back in the lab, we used a sensitive Mo/W determination method that we had developed based on adsorptive stripping voltammetry with a hanging mercury drop electrode [59], to measure that [WO_4_^2−^] = 0.03 μM and [MoO_4_^2−^] = 0.18 μM [60]. So apparently, local W was not much higher than Mo, but rather a bit less, and, therefore, a WtpA 650 times more selective for W would, under the circumstances, turn out to give the bug a mere 100 times preference for W over Mo uptake.

**Figure 8:**
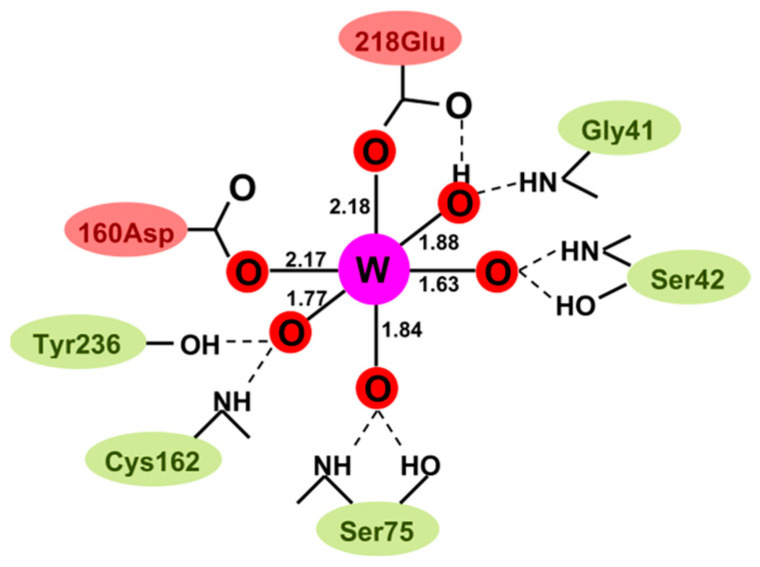
Schematic structure of tungstate bound by *Pyrococcus furiosus* tungsten transport protein A, WtpA. Upon binding of the tetrahedral WO_4_^2−^ oxoanion, the tungsten 4O coordination has been modified into a 6O deformed octahedron, with two coordination bonds to carboxylate side groups from amino acids [62]. The figure is from [63].

In an attempt to understand the W preference of WtpA on a molecular basis, we teamed up with Günter Schwarz from the University of Cologne, with whom Loes Bevers did a study in which WtpA was compared with *E. coli* ModA. It was already known from crystallography that the oxoanion binding sites in ModA and WtpA differ: the binding in ModA is “classical”, that is, the tetrahedral oxoanion MoO_4_^2−^ is bound by a number of hydrogen bonds to amino acid NH and OH groups [61]. Contrarily, in WtpA, WO_4_^2−^ is not only bound via hydrogen bonds, but there are also two direct, covalent bonds from carboxylato O in Asp160 and Glu218 to the metal W, all in all resulting in a distorted octahedral metal coordination [62], as schematically pictured in Figure 8.

The latter leads to the remarkable property that “molybdate” bound to WtpA can actually be reduced to Mo^5+^ and Mo^4+^ [63]. With isothermal titration calorimetry, Loes and Günter found that ModA had essentially identical K_D_ values for molybdate and tungstate; there was no selectivity. With WtpA, for which a K_D_ ratio of 650 had been previously established, mutation of Asp160 to Asn or Ala not only decreased the affinity but also completely abolished the selectivity [64]. One amino acid did it all! Of course, this is not yet a chemical explanation, but at least it is a molecular localization of the selectivity.

That the combined W/Mo preference factor of 100 for WtpA is indeed not the complete story of cellular selectivity became clear when graduate student Ana-Maria Sevchenco developed radioisotope based metallomics methodology to study metal insertion in proteins (see next section). In contrast to a previous failed attempt in Mike Adams’ group [65], Ana succeeded in incorporating Mo in all five known W-enzymes of *P. furiosus* [60]. However, to get a significant amount of Mo incorporated, it required much higher ratios of Mo over W in the medium than the natural habitat numbers quoted above. The highest incorporation was 0.9 Mo in FOR, using a 2000-fold excess of Mo over W in the medium. Moreover, the amount of incorporation differed for the five different W-enzymes from 0.14 to 0.90. These results suggest that metal selectivity is not limited to the level of import, but also occurs intracellularly, presumably during biosynthesis of the enzymes, in particular, during complex formation of the metal with the pterin ligands, in *P. furiosus* catalysed by the two paralog enzymes MoeA1 and MoeA2 [60,66].

## 10. Novel Systematics: The W Metallome

When, in the early years of this century, one could witness the blooming of many descendants of genomics, with a dedicated “omics” for just about every class of cellular molecules, we thought to perceive a neglect of the class of biological metal ions, and so we tried to design a metallomics. The term, in our approach, should not mean the definition of a research field, say, on the identification, distribution, dynamics, role, and impact of metals and metalloids in biological systems’, as implied in the name “Metallomics” of the journal that was established by the Royal Society of Chemistry in 2009 (in 2021 transferred to Oxford University Press), but rather should define a methodology to systematically identify the complete set of native metalloproteins for a given metal in a given cell. We quickly convinced ourselves that such a challenging task was only likely to be completed with the use of radioisotopes of the desired metal. This decision was probably helped considerably by the on-campus availability of a nuclear reactor for research purposes, which was populated, as it turned out, by helpful colleagues interested in our quest, such as Gerard Krijger and the head of the Department of Radiation, Radionuclides, and Reactors, Bert Wolterbeek. Within our own Department of Biotechnology, our project team was also extended by protein mass spectrometry specialists Martijn Pinkse and Peter Verhaert.

The principle we envisioned was simple: (1) add a specific metal radioisotope to the growth medium of a given organism; (2) separate the proteome in 2D gel electrophoresis; (3) localize the radioactivity; (4) wait for the radioactivity to decay; (5) identify the proteins in the previously radioactive spots. A practical waiting time for decay required a half-life roughly of the order of 24 h; hence, suitable isotopes were, e.g., ^56^Mn, ^65^Ni, ^64^Cu, ^67^Cu, ^69^Zn, ^99^Mo, and ^187^W [67] (we also developed a modified protocol for ^59^Fe with a half-life of 44.5 days [68]). After some heavy brain racking, we dubbed our method MIRAGE, which is an acronym for Metal Isotope Native Radioautography in Gel Electrophoresis. As it turned out, ^187^W proved to be the champion isotope for MIRAGE, with its 23.7 h half-life and its very high specific activity in bequerel per gram [69,70]. So, every reason exists to try the application of MIRAGE to the analysis of the tungstoproteome of *P. furiosus*.

**Figure 9:**
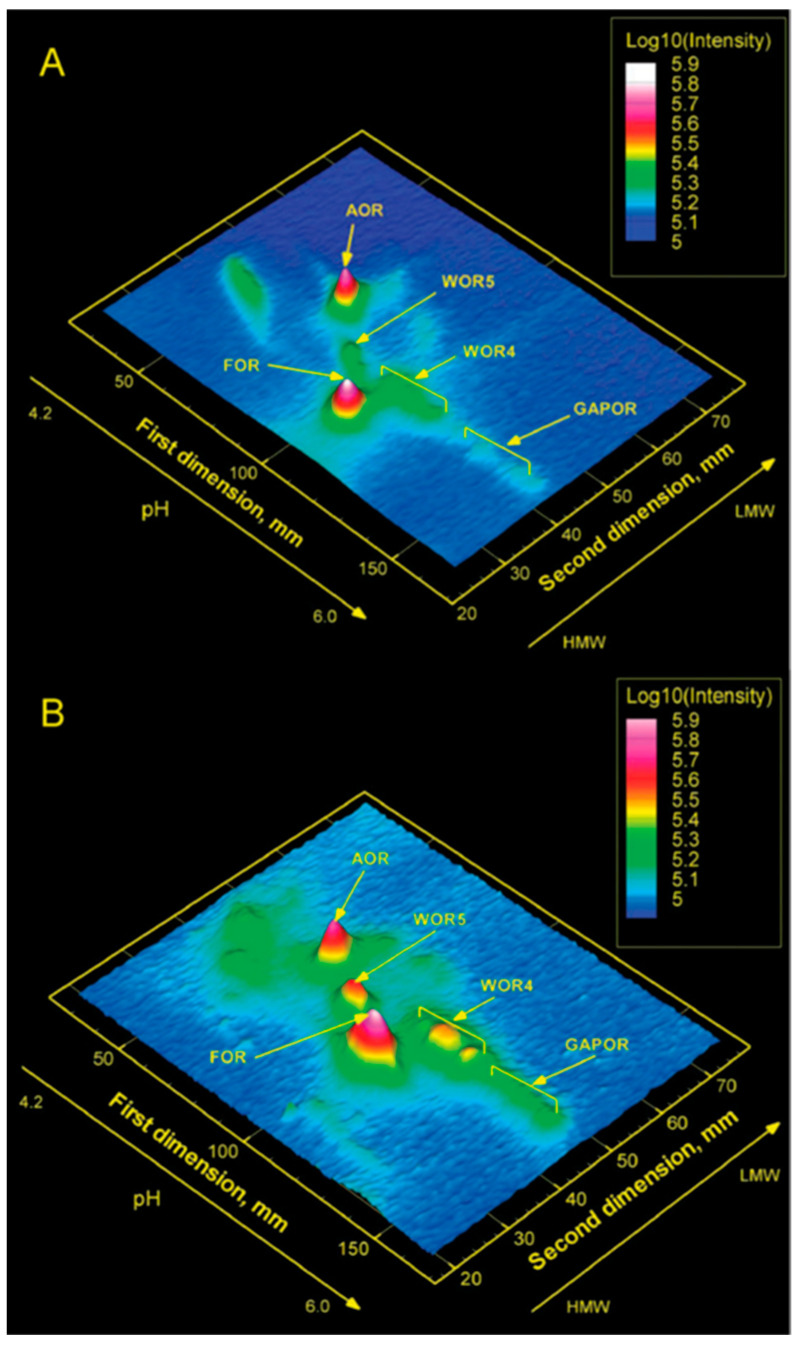
Tungsten metallomics of *Pyrococcus furiosus*. Cells were grown in the presence of radioisotope ^187^W at 95 °C (**A**) and, additionally, after a cold shock at 72 °C (**B**). All five known tungstoenzymes, and no others, are detected [69].

Figure 9 gives a graphical representation of W-MIRAGE for *P. furiosus* grown at 95 °C (top) and with additional growth at 72 °C (bottom). The latter condition was applied because a previous study by Mike Adams’ group had shown that “cold shock” (i.e., 72 °C) induced increased expression of some of the W-enzymes. The experiment is straightforwardly interpreted as showing that five, and not more than five, W-proteins are expressed, whereby each is identified in mass spectrometry as one of the known five AOR W-enzymes of *P. furiosus* [69]. In a subsequent study, we also found the proteins MoeA1, MoeA2, and MoaB, all involved in the biosynthesis of the tungstopterin cofactor [66], to coincide with radioactive peaks associated with specific AOR W-enzymes [60]. Furthermore, ^99^Mo containing WtpA was found in MIRAGE of the membrane fraction (presumably as WtpABC) when cells were grown on high Mo and very low W but not with high W, suggesting negative feedback control of tungstate concentration on the expression of WtpA [60]. All in all, it looks like all tungsten proteins and tungsten-related proteins have been identified in the tungstoproteome of the model organism *P. furiosus*.

## 11. Concluding Remarks

This review is a personal, therefore biased, account of my tungsten biochemistry research activities. For a more general and balanced account of the field of tungsten enzymes, I take the liberty of referring to my earlier reviews [2,3,5,71]. Subsequent reviews on tungsten enzymes by other authors are available as well [72,73,74].

It is time to draw up the balance sheet: what has been achieved and what remains to be discovered? The tungstobiochemistry research community can now pick from a nice, and sometimes even close to comprehensive, butterfly collection of W-proteins and crystallographic structures to work on. We asked the question “why tungsten now”, and we have a qualitative answer: reduction potentials of W versus Mo present an advantage when the cell is after reactions at low potential and/or after the reductive version of a reaction. On the other hand, environmental abundance of W over Mo does not seem to answer the “why” question to any significant extent, as cells can be extremely efficient in thermodynamically uphill internalizing W. Additionally, for a more detailed, quantitative answer in terms of the local enzymology of the tungstopterin cofactor, experimental data are scarce. For example, we have no indication to support the proposal by theoretical chemists that pterin may participate in radical redox catalysis. The key problem now appears to be one of time resolution. Steady-state-kinetics does not resolve reaction intermediates. Furthermore, the entanglement of different time scales in the kinetics of mixtures of resting and active enzyme molecules leads us astray. So, except for emeriti writing historical reviews, there is no reason for idling: tungstoenzymology is still a largely undiscovered land.

## Figures and Tables

**Table 1:** Tungstoenzymes: reactions catalyzed, names of enzymes, and their abbreviations ^a^.

*R*CHO	+	H_2_O	→	*R*COOH	+	2[H]	aldehyde oxidoreductase (AOR)
C_6_H_7_(CO)-*CoA*			←	C_6_H_5_(CO)-*CoA*	+	2[H]	benzoyl-CoA ^b^ reductase (BCR)
HCOO^−^ + H^+^			⇆	CO_2_	+	2[H]	formate dehydrogenase (FDH)
*MFR*-CHO	+	H_2_O	←	*MFR* + CO_2_	+	2[H]	formyl-MFR ^c^ dehydrogenase (FMD)
C_2_H_2_	+	H_2_O	→	CH_3_CHO			acetylene hydratase (ACH)
(CH_3_)_2_S	+	H_2_O	←	(CH_3_)_2_SO	+	2[H]	dimethylsulfoxide reductase (DMSOR)
NO_2_^−^	+	H_2_O	←	NO_3_^−^	+	2[H]	nitrate reductase (NAR)

^a^ Table modified from [5]. The format of the table is intended to emphasize general correspondences between reactions catalyzed by tungsten enzymes, namely, the use of water as a co-substrate and the occurrence of two reducing equivalents. This requires writing some of the reactions from right to left. ^b^ CoA is coenzyme A. ^c^ MFR is methanofuran.

## Data Availability

No new data were generated for this review.
